# The employment preferences of young people in Canada: a discrete choice experiment

**DOI:** 10.1186/s12889-025-21515-y

**Published:** 2025-02-21

**Authors:** Roberta L. Woodgate, Corinne A. Isaak, Julia Witt, Pauline Tennent, Ashley Bell

**Affiliations:** 1 College of Nursing, Rady Faculty of Health Sciences, University of Manitoba, 89 Curry Pl, Winnipeg, MB R3T 2N2 Canada; 2 Department of Economics, Faculty of Arts, University of Manitoba, 15 Chancellors Circle, Winnipeg, MB R3T 2N2 Canada; 3 Centre for Human Rights Research, Faculty of Law, University of Manitoba, 442 Robson Hall, Winnipeg, MB R3T 2N2 Canada

**Keywords:** Young workers, Precarious employment, COVID-19, Pandemic, Discrete choice experiment, Canada

## Abstract

**Background:**

Young people across the world are facing numerous challenges, with unemployment and precarious employment being substantial issues, impacting young people with all levels of education. For many young people, the pandemic exacerbated their employment precarity. While efforts were made to ameliorate these pandemic related challenges for young people, information about the employment preferences of Canadian young workers (YW) is limited. The aim of this study was to understand the employment needs, challenges and preferences of Canadian YW in the COVID-19 era and beyond.

**Methods:**

Using discrete choice experiment, YW from across Canada aged 18–29 years old were recruited to participate in an online survey October 2022 to April 2023 which was offered in both English and French. Nine job attributes were identified based on findings from the qualitative component of this mixed methods project: wage, earnings stability, job flexibility, vacation, sick time, health insurance, and workplace policies (respectful workplace, and being valued and understood as an employee). Respondents were presented with nine choice sets, each representing two scenarios that differ on policies or actions (attributes) related to their employment during the COVID-19 pandemic.

**Results:**

Based on the respondent (*N* = 231) sample, analysis revealed that of YW aged 18–29 years, most valued having employment benefits along with workplace policies. These values were strongest for women and 18–21-year-olds. Overall, the employment preferences of Canadian YW in the current study align with four of five attributes considered by the International Labour Organization as minimum standards for decent work. These include adequate compensation, adequate access to health care, adequate free time and rest, and organizational values that support one’s [own and] family values. More specifically, study findings show that within the cohort there are strong gendered and aged-based preferences for non-monetary over monetary job attributes. These include employment benefits along with equitable, supportive employment policies.

**Conclusions:**

The findings suggest that health and wellbeing are highly valued by YW and are among key drivers of employment preferences for Canadian YW during and after the pandemic, and therefore call for policies in the workplace that support the health and well-being of YW.

**Supplementary Information:**

The online version contains supplementary material available at 10.1186/s12889-025-21515-y.

## Background

According to the United Nations (UN) there are currently 1.2 billion young people aged 15 to 24 years, accounting for 16% of the global population [103]. In Canada, youth aged 15 to 29 years old form approximately 19% (7 million) of the country’s population [38, 86]. The COVID-19 pandemic had a significant impact on this demographic, particularly regarding their employment. The Director-General of the International Labour Organization (ILO) asserted that the COVID-19 pandemic was not only a global health crisis but “also a major labour market…crisis that is having a huge impact on people.” [46] Young people, frequently working in precarious employment, frontline and low income positions (i.e. cashiers, merchandisers, delivery drivers), were among those providing essential services during COVID-times. Simultaneously, young people often have limited social security, income, or agency to advocate for their rights [7]. For many young people, the pandemic exacerbated their employment precarity. While efforts were made to ameliorate these pandemic related challenges for young people (i.e. wage subsidies), information about the employment preferences of Canadian young people is limited.

### Youth employment

Young people across the world are facing numerous challenges, with unemployment and precarious employment being substantial issues, impacting young people with all levels of education [98]. In Canada it was estimated that in 2022 11% of 15–29 year olds were ‘not in education, employment or training’ (often referred to as NEET) [93]. Recent Labour Force Survey (LFS) estimates *released by Statistics Canada* indicate that the employment rate 15 to 24 year olds continues to decline since April 2023, falling 4.4 percentage points to 54% in June 2024. For this same age group, in June 2024, the employment rate for returning students was 46.8%, its lowest since June 1998, aside from June 2020 the first year of the pandemic [94].

Although a global definition of precarious employment (PE) has not been determined [8], the ILO defines PE using terms such as “low pay”, “lack of benefits”, “uncertainty” and “ambiguous” (p. 30) [45]. The increase of PE is due in large part to greater use of flexible work such as contract, term, part-time, or staff-for-hire (‘gig’) positions by organizations and businesses [49, 99]. Youth and informal workers in particular are exposed to greater levels of PE [39] as these flexible positions are frequently filled by young people. Furthermore, experts predict that changes in labour market structures due to technology will see many jobs being replaced with automation, potentially creating inequalities especially for young people as they are in labour market entry and early career stages [71]. With PE rapidly becoming the new norm, economists are predicting a potential lifetime financial impact for this young cohort [7, 43].

In addition to economic impacts, precarious employment has been linked to deteriorating lifestyle habits, and delays in the ability to progress into adulthood [98] and NEET status is correlated with low socioeconomic status, lower educational attainment, young parenthood and more precarious housing [6, 23, 27, 30, 41]. Further, greater exposure to PE for young people unfairly increases the risk of health issues [39], particularly for mental health (MH) issues [98, 105]. Globally, mental illness impacts approximately 25% of young people at some point in their lives [32], and “the global economic and societal burden of mental health disorders for this age group is rising at an alarming rate” (p. 1) [32]. In Canada, 15–24 year olds are more likely to experience mental illness and or substance use disorders compared to any other age group [112].

Compounding these employment, economic and health circumstances, young people face additional challenges in their workplaces. According to the ILO definition of decent work there are minimum standards required to adequately function at work; including 1) physical and psychological safety, 2) adequate access to health care, 3) adequate compensation, 4) adequate free time and rest, and 5) organizational values that support one’s [own and] family values [24]. However, these workplace standards are not always implemented within young peoples’ work environments. For example, in the Canadian labour market and workplace, discrimination and racism are significant barriers to employment and advancement [35] particularly for newcomer, Black, Indigenous, and People of Colour, and 2SLGBTQI + youth who often experience racism and discrimination during hiring [18], and entrapment in precarious jobs [10].

Occupational health and safety guidelines advise young people to know their workplace and employment rights [12], yet the precariousness of employment makes it increasingly difficult for them to speak up and have a sense of agency over their work experience. Confusing and ambiguous guidelines also make it challenging for youth to know what their rights are. For instance, young people may not have been informed or be aware of whether their position is union-based or comes with health benefits or paid sick time. Furthermore, given the high number of young people in PE (e.g., contract, part-time, or term positions), many may not have workplace health benefits, discrimination policies, or labour legislation.

### Impact of the COVID-19 pandemic on young workers

With the pre-COVID state of precarious employment and skyrocketing unemployment rates in Canada [88], the COVID-19 pandemic had significant detrimental impacts on the lives and employment of Canadian young workers (YW), including significant reductions in work hours [87] or job losses [61, 84]. This was especially true during the first year of the pandemic when jobs in the food, accommodation and tourism industries were among the hardest hit [97], a sector wherein nearly half (48.15%; 977,050) of the employed (*N*= 2,029,335) Canadian 15–24-year-olds were working in May 2021 [70].

Adding to these barriers, the pandemic, had the greatest effect on people who experience discrimination [72], with inequality and marginality more visible, severely impacting employment for some YW [17, 58]. For instance, loss of work hours was more likely to have been experienced for Canadian YW who were living with a disability (34%) or Indigenous-identifying (particularly First Nations) (45%) [73].

Given the pre-pandemic status of mental illness among Canadian young people [112], COVID-19 has served to further deteriorate their mental health (MH) and exacerbate pre-existing MH disparities [95]. For example, more young people experienced symptoms of depression, anxiety, stress, and worry, as well as an increase in the severity of these symptoms [34, 57, 83, 106], as well as increased substances use such as alcohol and cannabis [83]. These MH concerns were amplified for racialized young Canadians [13], for essential and frontline workers [15, 48, 109] as well as those with financial concerns, and those who were not working/laid off during COVID-times [83, 106] Essential and frontline workers, also noted greater stress and worry associated with contracting and transmitting COVID-19 and accessing proper personal protective equipment [15, 29, 33, 109].

### Engaging young workers

Young people today have tremendous potential to be active partners in global society [47]. Indeed, societies depend on young workers to develop and support future economies, particularly with the impact of technological advancements and globalization on work practices [36]. In 2018, the Government of Canada engaged 1000’s of youth from across the country to develop Canada’s first youth policy, learning “that young people want to have a say in decisions that affect their lives and impact their personal outcomes” (p. 5) [37]. Given the impact of the COVID-19 pandemic on young workers and the ongoing challenges to the labour market from COVID-19, the ILO contends that it is crucial that we engage in social dialogue, working with employees as a means to build public trust and garner support for measures that are needed to overcome the crisis [46]. These circumstances point toward the need for engagement with Canadian young people to ascertain their preferences related to employment.

In the context of competing demands, insufficient resources and the recent COVID-19 pandemic, the objective of this study was to engage with Canadian young people to detail their employment preferences to develop priority-setting policy recommendations to improve employment outcomes and workplace environments for YW.

## Methods

This study is part of a mixed methods research project to understand the employment needs, challenges and preferences of young people in the COVID-19 era and beyond. Prior to conducting the DCE survey, qualitative interviews and a graphic recording focus group were conducted from October 2020 to April 2022 with 33 YW aged 18–26 in the Canadian province of Manitoba, who had worked a minimum of 30 h per week prior to COVID-19 onset and were living independent of their parents [108]. Units of meaning were delineated from the interview and focus group data, clustered to form thematic statements and themes were then extracted. These themes and findings from the interviews and focus group helped to inform the DCE survey design. Additional details regarding the methods for the DCE component are provided below.

### Recruitment

With an aim to engage a diverse demographic from various geographical areas, we used a multi-modal recruitment approach, which balances pros and cons of individual recruitment methods and improves access to recruitment materials [59]. Recruitment was ongoing beginning in October 2022 to April 2023 and all materials were available in both English and French. An email script describing the study details along with study flyers with the online survey link and QR code were sent to 61 youth-serving organizations, universities, and youth employment agencies across Canada, requesting organizations to share the study information with 18–29-year-old youth accessing their services, or via their distribution lists. Social media was also used e.g., the principal investigator’s (first author) research program social media platforms (e.g. Facebook), including two 7-day paid Instagram ads with set boundaries (e.g. only Canadian; ages 18–29).

The anonymous online survey (see Additional File 1) was offered in both English and French and administered via Qualtrics software(60). Potential participants were informed in the consent form that “Participation in the study is completely voluntary and you can withdraw at any time with no impact to your education, grades, relationships with your teacher/school/youth leader/mentor, or employment”. Validation of responses strategies included bot detection (reCAPTCHA) [81] at the beginning of the consent/survey, and review by research team members of several open-ended question response combinations (e.g. survey Q9—work status, e.g. full time/casual) and Q10—# hours working per week). Suspected fraudulent responses were removed and not included in analysis.

Following provision of informed consent, the software directed respondents to the survey, which included demographic questions, a section on Job Satisfaction and Attitudes to work, preferences for types of jobs (Discrete Choice Experiment (DCE), questions on earnings and benefits, and an optional open-ended question for additional comments about participants’ work. This article focuses on the DCE findings.

### DCE Design

Discrete Choice Experiments (DCE’s) are a quantitative technique initially used in marketing, and increasingly in health economics to address policy-related concerns [16], as well as with young people to understand their needs in the labour market [26, 67]. DCE’s are a useful instrument for quantifying preferences and priority-setting in the context of competing demands and insufficient resources [21, 101]. They are able to quantify the implicit trade-offs made by respondents and can be used to calculate ‘willingness-to-pay’ and other measures, making recommendations from DCEs precise and responsive.

Discrete choice experiment design and sample size were calculated following completion of the qualitative interview segment of the project, which informed the nature and number of attributes and levels we wanted to include [108] (and thus parameters to be estimated) to focus on youth’s needs and opinions. For example, key qualitative themes included workplace legislation (sick days/leave, salary/wages), workers’ rights, and workplace environment and employment practices. Discussion amongst authors (RW, CI, JW, AB) and searching for attributes to align with these were then discussed and determined among authors and thus focused on labour policies during the pandemic and health-related issues to better understand YW preferences during COVID-19 and potential future health crises. A review of the literature was then conducted as well as online searches for workplace policies and union agreements to determine definitions and levels for each potential attribute [9, 50, 55, 80, 100]. Authors acknowledge that the literature on attributes for job preferences is broad, and includes other attributes such as workload, control over work [69], as well as support for MH and wellness in the workplace, life skills, and career counselling [77]. Changes from early to final versions of the DCE were discussed and decided on by the authors and focused primarily on levels. For example, Hourly Earnings Changes (10% increase; no change, 10% decrease), and Job Flexibility Type (time, duration, location) were changed to Hourly Wage Changes (30% higher than provincial minimum wage; Provincial minimum wage; 15% higher than provincial minimum wage, and Job Flexibility Type (Weekdays Day and Evening; Evenings and Weekends; Rotating shifts; Weekdays daytime only). The final version of attributes and levels are listed below in Table 1. The DCE was generated with SAS software [56] and based on D-efficiency [52]. The discrete choice experiment (DCE) included 7 attributes, one with 4 levels, and 6 with three levels. SAS software was used to generate a fractional factorial design with 36 choice sets blocked into 4 groups of 9 using the SAS %mktblock macro. Youth were presented with 9 choice sets, each representing two scenarios (alternatives) that differ on policies or actions (attributes) related to their employment during the pandemic (i.e., employment circumstances that youth felt exposed them to risks; changes or accommodations that were made in response to the pandemic that they felt improved their safety; and changes they would like implemented to make their work safer). Respondents were asked “Which job do you think is better?” and chose the option they liked best from each choice set. Through a series of choices, relative preferences for all attributes were captured and the results of the data analyses provided a relative ranking of all attribute levels in the DCE.

**Table 1:** Discrete Choice Experiment Attributes & Levels

Attribute	Levels
Wage (Hourly pay in CAD dollars/hour)	30% higher than provincial min. wage
	15% higher than provincial min. wage
	Provincial minimum wage
Stability (Earnings stability type)	Hourly wage plus bonus based on profits and/or performance
	Fixed wage, but contract can end any time
	Fixed wage for a given period of time
Flexibility (An employee’s ability to determine when they work and their schedule)	Weekdays daytime only
	Weekdays day and evening
	Evenings and weekends
	Rotating shifts
Vacation (Paid days provided by employer to be used for personal time off (in number of days per year)	21 days per year
	14 days per year
	No paid vacation time
Sick Time (Paid leave used when unable to perform working duties, not because of injury/illness on the job. Includes mental and physical illnesses)	1.5 days per month
	1.0 days per month
	No paid sick leave
Health Insurance (Non-monetary compensation provided beyond employee, paid by the employer)	Extended (80% of expenses incl. drugs, vision, dental)
	Basic (50% of expenses incl. drugs)
	None
Workplace Policies (psychosocial workplace environment, practices and policies)	Respectful Workplace Policy (non-discriminatory, non-racist, etc.)
	Being valued and understood as an employee (ability to provide input, concerns are acknowledged and acted upon by supervisors)
	None

### Analysis

Data was analyzed with NLogit software [68] (multinomial choice data specialized software) using a mixed logit (or random parameters logit) model with all attributes except wage specified as random parameters (normally distributed) to allow individual heterogeneity [82]. Results were computed using 500 Halton draws. Alternative-specific constants were included in each regression, and all attributes and levels were effects coded. Effects coding follows a (−1, 0, 1) notation, in which the base category is defined by −1’s. This permits main effects to be interpreted independently from the level of the other variables [42].

### Ethical considerations

Ethical approval was obtained from the Ethical approval was obtained from the University of Manitoba Fort Garry Research Ethics Board 1. An informed consent was included within the Qualtrics platform for which all participants provided informed consented to prior to proceeding with survey completion. Participation was voluntary thus respondents were not paid for completing the survey. In recognition of their time and input, respondents were given the opportunity to submit their email address through a separate link via Qualtrics, to enter a draw to win one of three $100 gift cards of their choice (e.g., Amazon, Walmart). After the online survey closed, a random number generator process was used to draw three respondents who had provided their names and contact information for this purpose separately from their DCE survey responses. $100 gift cards were then provided electronically to each of the three winners.

## Results

### Sample

Participants demographics are listed below in Table 2. Of the 231 respondents, most respondents (77.5%) identified as women, while half (50.0%) were in the youngest 18–21-year-old age group, referred to going forward as “younger workers”. Three fourths (76.2%) of respondents had at least some college/university or college diplomas/undergraduate degrees. Nearly half (41.7%) worked in the food or retail employment sectors, and only one fourth (24.7%) indicated they currently received employment benefits.

**Table 2:** Participant Demographics

		Number	Percent
Gender	Woman	179	77.5
	Man	32	13.8
	Another gender	20	8.7
Age Group	18 – 21	116	50
	22 – 25	76	32.9
	26 – 29	39	16.8
Province	AB	24	10.3
	BC	26	11.2
	MB	21	9.1
	NB	2	0.9
	NL	1	0.4
	NWT	3	1.3
	NS	3	1.3
	ON	138	59.5
	QC	7	3.0
	SK	6	2.6
	YK	1	0.4
Education	Less than high school	4	1.7
	High school diploma	50	21.5
	Some post-secondary	96	41.3
	College diploma	13	5.6
	University degree	68	29.3
Citizenship^a^	Canada	201	87.0
	Other	27	11.7
Indigenous^a^	Yes	18	7.8
	No	213	91.8
Sector^a, b^	Education	56	22.7
	Food	54	21.9
	Healthcare	30	12.1
	Non-profit	20	8.1
	Retail	49	19.8
	Transport	3	1.2
	Finance	9	3.6
	Engineering	4	1.6
	HR/Marketing	8	3.2
	Technology	2	0.8
	Security	3	1.2
	Arts	4	1.6
	Manufacturing	3	1.2
	Unemployed	2	0.8
Ethnicity^a^	Asian	24	10.4
	SE Asian	21	9.1
	S Asian	37	16
	W Asia	9	3.9
	Black	13	5.6
	Caribbean	1	0.4
	Caucasian	79	34.2
	Latin American	5	2.2
Benefits^a^	Currently has some benefits	57	24.7
Part-time work^a^	20 h or less per week	138	59.7
Location^a^	Hybrid	39	17.1
	On-site	165	72.4
	Virtual	24	10.5
Wage^a^	Earns minimum wage	47	20.3

^a^ Not answered by all respondents; percent out of total, including missing responses

^b^ Some individuals checked off more than 1 sector

### Discrete choice experiment

The main results are from the main effects models (which include only attribute levels) and include mean and standard deviation estimates, as well as willingness to pay (WTP). WTP standardizes estimates by the wage coefficient and is comparable across attributes and models. The discussion of results, therefore, focuses on WTP. Below is an example of the effects coding and the WTP formula for the “Vacation” attribute, level “21 days per year” (${\beta }_{1}$), where “No paid vacation time” ($-{\beta }_{1}-{\beta }_{2}$) is the base category:

**Table:** 

	${\beta }_{1}$	${\beta }_{2}$
21 days per year	1	0
14 days per year	0	1
No paid vacation time	−1	−1

$${\text{WTP}}=\frac{{\beta }_{1}-(-{\beta }_{1}-{\beta }_{2})}{{\beta }_{\text{wage}}}=\frac{2{\beta }_{1}+{\beta }_{2}}{{\beta }_{\text{wage}}}$$

The WTP is interpreted as the amount that individuals, on average, are willing to pay per hour of work (or give up from their hourly wage) to have (if positive) or avoid having (if negative) that particular attribute level, relative to the base level.

#### Main effects model results

The main effects regression was run on several samples: the full model (includes all respondents); women; part-time workers; younger workers (age 18 – 21); BIPOC youth (e.g., Indigenous, Asian); respondents with more than high school education; and workers in the retail, food, transportation, manufacturing or security sectors. Models with smaller samples outperformed the full model analysis, based on pseudo R^2^, and the Akaike and Bayesian Information criteria.

**Fig. 1:**
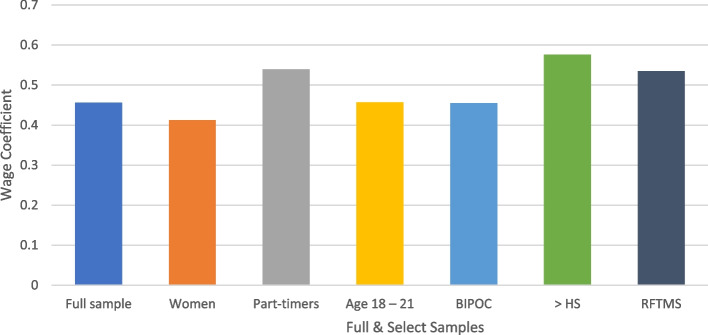
Wage coefficient for the full and select samples

Figure 1 shows the wage coefficient for the full and select samples. It was positive and significant at 1% across all models and did not vary much in magnitude (ranging from 0.4123 among women to 0.5761 among respondents with more than high school education), suggesting that respondents are more likely to choose a job with a higher wage, all else equal.

**Fig. 2:**
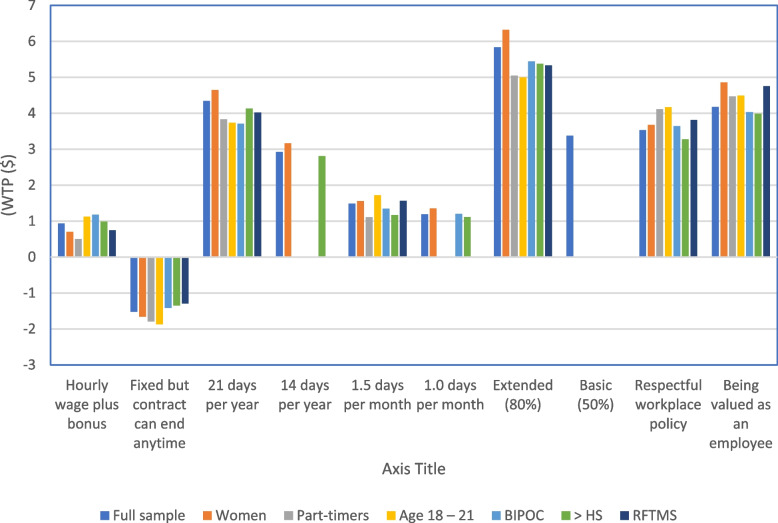
Willingness to pay (WTP) for the full and select samples. Notes: omitted bars represented attribute levels that are not statistically significantly different from base category

Figure 2 shows willingness to pay (WTP) for the full and select samples. The results were consistently positive and significant across all models for several attribute levels, though with varying magnitude: “hourly wage plus bonus”, “21 vacation days per year”, “1.5 sick days per month”, “extended (80%) insurance benefits”, “respectful workplace policy”, and “being valued as an employee” were positive and significant for all samples. Small positive effects were observed for “hourly wage plus bonus”, with BIPOC willing to pay the most ($1.18 per hour) and part-time workers willing to pay the lowest amount ($0.50 per hour); and for “1.5 days per month of sick leave”, with younger workers willing to pay the most ($1.72 per hour), and part-time workers willing to pay the lowest amount ($1.11 per hour). Fairly large positive effects were observed for “21 paid vacation days per year” (ranging from $3.71 per hour among BIPOC to $4.65 per hour among women); and for a “respectful workplace policy” (ranging from $4.17 per hour for younger workers to $3.28 per hour for respondents with more than high school education). The largest positive and significant values, suggesting these attributes are the most highly valued, were observed for “extended (80%) insurance benefits”, ranging from the highest WTP of any attribute level at $6.32 per hour for women, to $4.99 per hour for younger workers, and for workplace policies that promote “being valued as an employee” ranging from $4.86 per hour for women to $3.99 per hour for respondents with more than high school education.

The only attribute level that was negative and significant across all samples (and, in fact, the only negative WTP in all models) was for wage stability level “fixed, but contract that can end anytime”. The magnitude was small, ranging from $1.87 per hour for younger workers aged 18–21, to $1.30 per hour for workers in the retail, food, transportation, manufacturing and security sectors. A negative WTP means that respondents need to be compensated to accept this attribute level over the base (“fixed for a given period”). Please also see Appendix Table 1: Main Effects Models for Different Samples and Appendix Table 2: Willingness to Pay for Different Samples.

All levels of the “wage stability” attribute had small WTP’s, suggesting that preferences are not that strong compared to some of the other attributes. The most important attribute levels were extended health insurance; 21 paid vacation days per year; and some kind of workplace policy (both “respectful workplace policy” and “being valued as an employee” were preferred to no policy).

#### Interaction effects model results

The interaction models use the full sample and add one at a time to each regression. The socio-demographic variable is interacted with all attribute levels to explore heterogeneity in attribute level salience between socio-demographic groups.^1^ There are nine interaction variables, each is used in a separate model: part-time workers; younger workers (age 18 – 21); women; retail, food, transportation, manufacturing and security sector workers; BIPOC; high school highest level of education achieved; current job has work benefits; works on site only (versus hybrid or remote only); earns minimum wage; education, health, non-profit, technology sector workers. Models with interaction effects performed similar to the full analysis main effects model and were outperformed by the analyses using subsamples. The interaction effects model results are generally consistent with the results of the main effects analyses; significant interaction effects that indicate different preferences are briefly discussed below.

Younger workers’ (18–21-year-olds) willingness-to-pay is significant and the same sign compared to the main effects for “fixed wage, but contract can end anytime” and “respectful workplace policy”, but opposite in sign for “evening and weekend” and “weekdays only” shifts. Insignificant main effects were offset by significant interaction effects by workers in the education, health care, non-profit or technology sectors for “weekdays day & evening” shifts (negative WTP); minimum wage workers for “evening & weekend” shifts (positive WTP); individuals with less than high school education for “basic insurance” (positive WTP); and individuals in the retail, food, transportation, manufacturing or security sectors (RFTMS) had a positive WTP for “1.0 sick days per month”.

The main effects in the models with interactions are generally consistent with those of the main effects only analyses: positive and significant effects were found for all models for “hourly wage plus bonus”; “21 vacation days per year”; “extended health insurance”; “respectful workplace policy”; and “being valued as an employee”. “Fixed (wage) but contract can end anytime” was negative and significant across all models. “1.5 days paid sick leave per month” was not significant for the women interaction model but was significant and positive for all other interaction models and all main effects models. Similar to the main effects analyses, magnitudes were largest for “extended health insurance”, “21 vacation days per year”, “respectful workplace policy”, and “being valued as an employee”. Please also see Appendix Tables 3A and 3B: Interaction Effects Models, and Appendix Table 4: Willingness to Pay for Interaction Effect Models.

## Discussion

Being a young worker in today’s labour market comes with numerous challenges, particularly in the wake of the COVID-19 pandemic. Through the input of YW in Canada, this study provides new insights into their employment preferences and priorities which can be used to inform priority-setting to improve employment outcomes for this population. Aside from one recent Canadian study that used a DCE approach with young people who were not engaged in education, employment or training (NEET) to examine employment, education, and training preferences [40, 77], to the best of our knowledge, this is the first study to use a DCE approach to systematically explore the employment preferences of YW in Canada during COVID.

While our aim was to recruit a representative sample of YW from across Canada, our study sample did not fully attain this goal. For example, comparison of representation of YW in our sample across some provinces with the employment rate^2^of young Canadians aged 15–24 years old in January 2023 (recruitment mid-point) shows the following for Ontario (59.1% vs 56.6%), British Columbia (11.2% vs. 60.0%), and Manitoba (9.1% vs. 61.9%) [92]. Also, some provinces (Prince Edward Island) and territories (Yukon and Nunavut) are not represented in our sample which may lead to regional biases. In Canada, youth employment varies by education level. In our sample representation was, high school 21.5%, some post-secondary 41.3%, college diploma 5.6%, university degree (bachelor or higher) 29.3%. Whereas for of all 15- to 30-year-olds youth employed in Canada in 2019, the highest levels of education were, high school 64.3%, post-secondary certificate or diploma 82.8%, and bachelor's degree 83.4%, above bachelor’s degree 81.7% [63]. Also, more than half (59.7%) of YW in our sample worked part-time (20 h or less per week). While in January 2023, 44.56% of Canadian 15–24-year-olds in the labour force were employed part-time (< 30 h/week) [92]. The higher percentage of part-time workers in our sample may be due to pandemic employment markets or health-related restrictions. Then, given 77.5% of our sample identified as young women, this may explain the higher overall rate of part-time workers as Canadian young women are more likely to be employed part-time [85]. Also similar to higher participation of women-identifying participants in the current study, in a recent DCE study via online survey with Canadian 14–29-year-olds there were also higher participation of girls/women gender (53.28%), relative to boys/men (17.89%) or gender diverse (28.83%) [77]. Furthermore, for online surveys, previous research shows it is more common for women to respond than men, although the reason for these gender differences requires further research [4]. Although our study sample is not fully representative of YW across Canada, given this is the first to use a DCE approach to systematically explore the employment preferences of YW in Canada during COVID, is adds valuable contribution to the literature as well as information that can be used by employers both currently and during future health crises.

Overall, the employment preferences of Canadian YW in the current study align with four of five attributes considered by the ILO as minimum standards for decent work [44]. These include adequate compensation, adequate access to health care, adequate free time and rest, and organizational values that support one’s [own and] family values [24]. More specifically, study findings show that within the cohort there are strong gendered and aged-based preferences for non-monetary over monetary job attributes, including employment benefits along with equitable, supportive employment policies-discussed in more detail below. These findings suggest that health and wellbeing are highly valued by YW and are among key drivers of employment preferences for Canadian YW during and after the pandemic, and that there may also be a trade-off between non-monetary rewards and wages for YW [28].

As expected, Canadian YW prefer jobs with a higher wage. Although workers of all ages want fair pay [79] this finding aligns with results from the qualitative component of this study [108], as well as with DCE studies exploring employment preferences with young people from Switzerland [64] and Bangladesh for example [78]. Nonetheless, non-wage job attributes including employment benefits are components of decent work [24, 44] and play an even stronger role than wages in job preferences for Canadian YW, suggesting that these young people value work-life balance and have a quality of life mindset. This is particularly true for respondents who identified as women and or BIPOC (e.g., Indigenous, Asian) who are willing to pay (WTP) the most for “21 days vacation”. Similarly, “extended health insurance” is most valued by women followed by younger (18–21-year-old) workers. Many YW in the study sample are employed in the retail and food sectors, and fewer are in jobs with employment benefits which may have negatively impacted their health and wellbeing during COVID-19 and exacerbated pre-COVID vulnerabilities of this cohort. Thus, if given a choice, YW may be more likely to choose jobs with health insurance and vacation benefits included. Conversely, the finding that “basic insurance” is significant for YW with high school education or less but insignificant for all others may be related to differences in the types of jobs that are available to those without post-secondary education which are typically lower paying and without benefits. YW in these job types may not be aware of what the various levels of health insurance entail or may not be willing to give up the limited pay they do receive. Additional qualitative enquiry would be required to elucidate the reason for these findings.

Respectful workplace policies and policies that promote valuing and understanding of employees will be critical for retention of YW in post-COVID times. This is demonstrated in findings that YW in this study highly value having workplace policies (e.g., “respectful workplace policy” and “being valued as an employee”) over no workplace policies. These findings also align with decent work criteria [24, 44], and with findings from the qualitative findings of this study where participants recommended young people be respected and provided with opportunities to be heard in the workplace [108]. The only other Canadian DCE-based employment-related study with young people [40, 76] did not include values-based employment attributes, making this study a unique contribution to the Canadian literature. A DCE-based study with university students in Columbia, Ecuador, and Spain did include a working atmosphere attribute and work environment label (relationships with co-workers and supervisors), however, results specific to this label/level were not reported or discussed [62]. Employers would do well to take these preferences into consideration when developing compensation packages and when hiring YW as they have potential to positively impact employers’ abilities to recruit and retain the type of workers being sought [28].

Discussion of population group findings from the current study specific to gender- and race/ethnic-identity, and age is relevant here as it can contribute to recommendations for developing equitable work environments and opportunities for YW in Canada.

To begin, exploring gendered employment preferences, the only other study we are aware of that uses DCE to explore vacation benefits was conducted with a sample of young people in Kenya where the vacation attribute was a binary choice (yes/no), and both men and women favored jobs offering paid vacations [26]. Although in that study young men were willing to forgo slightly more wages for paid vacation than young women were. Previous DCE studies exploring preferences for health insurance as an employment benefits among engineering students at universities in Egypt and Indonesia [66] and among Kenyan youth [26] also reported preferences for better benefits including health insurance. Like results in the current study, both studies reported gender differences where young women were WTP more for health insurance than young men. Gendered preferences in job characteristics have been interpreted in previous studies as stemming from traditional gender roles and socialization [3, 96]. According to this interpretation, “men value high pay more than women, who consider other aspects of a job more important” (p. 3) [104]. This may also be the case for the current study findings; however, additional research would be required to verify this. Regarding gender-based effects, similar to our findings where workplace policies were highly valued among women YW, Perez-Carbonell and Santana reported that female university students in Spain value employment environments in which they are treated well by colleagues [74]. This characteristic has been explained as potentially due to women’s ‘desire for social recognition’ [74]. Gender-based discrimination in the Canadian work-place such as the gender-pay gap which starts from an early age [65], access to employment [54], and harassment [89] are also likely to influence young women’s preferences for respectful workplace policies. According to a 2019 Statistics Canada survey with post-secondary students, 20% of women students indicated they personally experienced discrimination in past year compared to 13% of men students [11]. In the same year, despite being more highly educated, young women earned approximately 6% less than young men [63]. Addressing these gender-based challenges requires government support and action on commitments within the Convention on the Elimination of All Forms of Discrimination Against Women (CEDAW) [31, 102]. By applying principles of equality to eliminate gender discrimination young women can reach economic success and further strengthen Canada’s economy [37], and empower them in their workplaces.

Our finding that BIPOC (e.g., Indigenous, Asian) respondents highly valued “21 days of vacation” in the aftermath of COVID-19 was not surprising as BIPOC individuals tend to have higher rates of employment in frontline and essential positions [75] and therefore may have been expected to work additional hours during the pandemic. Moreover, given mental health concerns were amplified for racialized young Canadians during the pandemic [13] who were also at greater risk of exposure to and contracting COVID-19 [53, 75], more vacation days is likely to be seen as desirable by this group. Discrimination and racism are also significant barriers to employment and advancement in the Canadian labour market [35], particularly for newcomer, BIPOC and 2SLGBTQIA + young people who often experience these issues during hiring [18]. These findings have implications for employers to ensure equity, diversity, and inclusion (EDI +) practices and policies are implemented in workplaces particularly for racialized YW in Canada.

According to the Statistics Canada report *A Profile of Canadian Youth,*“today’s youth are unlike any generation before” [86]. For example, Randstad Canada reported that 80% of Gen Z young people prioritize finding a job that aligns with their values and interests, compared to 59% of Millennials.(79) Given most (82.9%) of the study sample were aged 18–25 in 2023 the year of data collection, and fit within Statistics Canada age ranges for the cohort referred to as Generation Z (Gen Z) (born between 1997 and 2012) [90], further discussion of characteristics of Gen Zers is relevant here as it may influence employment and life-style preferences of YW that could inform employers’ hiring and workplace practices and policies going forward. Also, Generation Z makes up a considerable portion of the working-age population, is more diverse and more educated than former generations, and grew up with technology which has significantly impacted their lifestyles and values [22, 90]. Many of these YW [Gen Zers] are just entering the workforce and have specific values and expectations, for example, “… they want benefits packages that will help them navigate the day-to-day stress of modern living” [60], they value self-care [51], empathy from employers [25], supervisor support and positive coworker relations [1], and are anticipated to reshape future workplaces and the labour market [2, 90]. Likewise, results from a 2022 survey with Gen Z students at the University of Waterloo in Ontario, Canada show that work-life balance and good benefits including healthcare were highly rated by 83.4% and 76.1% of Gen Z workers respectively [110]. In order for young people to successfully move through life course milestones such as adulthood and marriage [14, 19] and to support future economies, both employers and government would do well to heed YW’ priorities and preferences in jobs typically held by YW. 

### Strengths, Limitations, and Future Research

A strength of the study is that we engaged with young people from across Canada who are experts in their own needs and employment preferences. This study is also the first to use DCE methodology to ascertain employment preferences of Canadian YW. Our study also included values based DCE attributes which is unique and aligns with the importance of lifestyle and values of today’s YW.

The study has limitations in that it used a non-randomized sample of Canadian young people who received the project recruitment information from a youth-serving agency, via Instagram, or the PI’s research program social media platforms and thus may not be representative of Canadian YW. The underrepresentation of YW respondents from some provinces is also a limitation and may create regional bias in our findings, limiting our ability to generalize findings nationally. Thus, the employment preference findings may be concentrated in specific regions of Canada and only pertain to these areas of Canada. Also, only (13.8%) male/man-identifying young people completed the survey which would not be representative of male YW in Canada which was approximately 58% during the study period [91]. We note here that previous research describes the gender effect in survey participation as a social phenomenon where women are more likely to participate [4, 5, 20]. As noted above, this is particularly observed with online surveys where it is more common for women to respond than men, although the reason for these gender differences requires further research [4]. Another limitation is the finding of no difference between ‘no health insurance’ and ‘basic insurance’ which may be due to wording of these options in the DCE survey, or to young workers’ experience with basic insurance being insufficient for their needs, thus favoring extended health insurance. These attribute levels may require better explanations of terms e.g., ‘benefits’ and what is typically included in a benefits package.

The authors recommend replicating this study in five years with the same or different age groups and a gender diverse and representative sample of YW in Canada to determine if employment preferences changed over time or are unique to certain groups of YW. Analysis and publication of findings from the survey sections not covered in this article including Satisfaction and Attitudes to work, questions on earnings and benefits amounts, and responses to the optional open-ended question regarding participants’ work would also provide important policy-relevant information. Moreover, similar to Wiswall et al. [107], a comparison of respondent job preferences with actual employment earnings and benefits, and job satisfaction would further enhance understanding of how preferences relate to employment outcomes.

## Conclusions

YW are set to become the future work force in Canada, key economic contributors, future leaders, and parents. Findings from this study are particularly salient as women, BIPOC, and young people are part of known marginalized groups within social and workplace environments. It is thus imperative that decent work policies are implemented and that the Convention on the Elimination of All Forms of Discrimination Against Women (CEDAW) [102] and Canada’s Youth Policy are acted upon, respecting youth voices and treating young people as equal members of society(52) to fulfill YW’ preferences for respectful workplace policies and being valued as an employee. These measures can provide all young people in Canada with the supports and opportunities required to succeed and thrive and leave a legacy for the generations that follow.

## Supplementary Information

Supplementary Material 1.

Supplementary Material 2.

## Data Availability

The datasets generated and/or analyzed during the current study are not publicly available owing to restrictions in the ethical approval of this study, but are available from the corresponding author on reasonable request.

## Abbreviations

ILO: International Labour Organization
NEET: Not in Education, Employment or Training
LFS: Recent Labour Force Survey
PE: Precarious Employment
MH: Mental Health
YW: Young Workers
DCE: Discrete Choice Experiment
BIPOC: Black, Indigenous, and People of Colour
WTP: Willingness to Pay
RFTMS: Retail, Food, Transportation, Manufacturing or Security Sectors
CEDAW: Elimination of All Forms of Discrimination Against Women

## Footnotes

1 Latent Class analyses were attempted but always yielded one class with large standard errors.

2 The employment rate for a particular group (age, sex, marital status, etc.) is the number employed in that group expressed as a percentage of the population for that group.
