# Supplementary material for: The employment preferences of young people in Canada: a discrete choice experiment
Source: BMC Public Health. 2025 Feb 21;25:715. doi: 10.1186/s12889-025-21515-y (PMC11844131; doi:10.1186/s12889-025-21515-y)
Supplement: Supplementary file 1 — Supplementary Material 1. [file 12889_2025_21515_MOESM1_ESM.docx]

**Young Workers**

**Discrete Choice Experiment**

**SURVEY1**

**For Young People 18-29 years old**

**PART A: Demographics**

1. **What is your age (years)?**

18-21
22-25
26-29

1. **What is your gender identity?** (Please check all that apply)

Agender

Woman

Man

Non-binary

Trans

Two-Spirit

Another gender or an additional gender identity (specify): __________
Prefer not to answer

1. **Which province/territory do you currently live in? [ADD list]**

Alberta, British Columbia, Manitoba, New Brunswick, Newfoundland & Labrador, Northwest Territories, Nova Scotia, Nunavut, Ontario, Quebec, Prince Edward Island, Saskatchewan, Yukon

1. **What is your highest level of education?**

Grade 8 or less Some university
Some high school University degree (e.g., Bachelor, Master’s, PhD)
High school diploma Other
Some college Prefer not to answer
College certificate/diploma

1. **In which year did you graduate from high school?**Year___________________ Not Applicable________________ _________________
2. **Of what country/countries are you a citizen (select all that apply)?**Canada, by birth
   Canada by naturalization (immigrant that’s been granted citizenship of Canada)
   Other country (please specify): _____________________
   Don’t know
   Prefer not to answer
3. **Are you now, or have you ever been, a landed immigrant? (a person granted the right to live in Canada permanently).**Yes
   No
   Don’t Know
   Prefer not to answer

**8a. Do you identify as an Indigenous person, that is, First Nations (North American Indian), Métis or Inuk (Inuit)?**Yes, First Nations (North American Indian)
Yes, Métis
Yes, Inuk (Inuit)
No, I do not identify as an Indigenous person

Don’t know

Prefer not to answer

**8b.** People living in Canada come from many different cultural and racial backgrounds. And individuals who identify as Indigenous may also acknowledge other cultural and racial backgrounds. Do you identify as… **.** SELECT ALL THAT APPLY

Caucasian, White or European - Please specify (e.g., British, French, Italian, Portuguese, German, Ukrainian, Russian): _________

Black - Please specify (e.g., Ghanian, Eritrean, Kenyan, Caribbean, North American): _________

Asian – Please specify (e.g., Chinese, Korean, Japanese. Taiwanese) _____________

South Asian - Please specify (e.g., Indian, Pakistani, Sri Lankan): _______________

Southeast Asian - Please specify (e.g., Vietnamese, Cambodian, Indonesian, Laotian, Filipino):_

West Asian - Please specify (e.g., Egyptian, Saudi Arabian, Syrian, Iranian, Iraqi, Israeli, Lebanese, Afghani, Palestinian):
_____________
Latin American, Central American, South American - Please specify (e.g., Mexican, Brazilian, Chilean, Guatemalan, Venezuelan, Colombian, Argentinian, Salvadorian, Costa Rican): _____

Caribbean (non-black)

Not listed (please specify): ____________

Don’t know

Prefer not to answer

1. **What job sector(s) are you currently employed in?** Retail (e.g., clothing, home improvement, grocery, drug store)
    Food & Hospitality (e.g., restaurant, catering, hotel)
    Healthcare (e.g., licensed practical nurse, healthcare aid, nurse)
    Education (e.g., educational assistant, librarian, research assistant)
    Transportation (e.g., (taxi, school bus, trucking)
    Non-Profit (e.g., community-based agency)
    Other (please provide) _________________________________
2. **What is your current work status?** (Please check all that apply)

Full Time Permanent
Part Time Term
Casual Contract
Temporary

1. **How many hours per week do you work in total?**5-10 11-15 16-20 21-30 31-40 41+
2. **Province/Territory currently working in**

Alberta, British Columbia, Manitoba, New Brunswick, Newfoundland & Labrador, Northwest Territories, Nova Scotia, Nunavut, Ontario, Quebec, Prince Edward Island, Saskatchewan, Yukon

1. **Work Location**

On-site Virtual Hybrid (some on-site, some virtual)

**PART B: Job Satisfaction and Attitudes to work**

The following questions refer to your MAIN job. Your MAIN job is the EMPLOYER who you work the MOST HOURS for.

**14. Please indicate how satisfied or dissatisfied you are with each of the various aspects of your MAIN job.**

|  | **Very Satisfied** | **Moderately satisfied** | **Neutral** | **Moderately dissatisfied** | **Very dissatisfied** | **N/A** |
| --- | --- | --- | --- | --- | --- | --- |
| Working conditions |  |  |  |  |  |  |
| Amount of autonomy/ independence |  |  |  |  |  |  |
| Your colleagues and fellow workers |  |  |  |  |  |  |
| Recognition you get for good work |  |  |  |  |  |  |
| Amount of responsibility you are given |  |  |  |  |  |  |
| Your hourly wage |  |  |  |  |  |  |
| Amount of challenge in your work |  |  |  |  |  |  |
| Your hours of work |  |  |  |  |  |  |
| Amount of variety in your job |  |  |  |  |  |  |
| Taking everything into consideration,  how do you feel about your job? |  |  |  |  |  |  |

**15. Formal processes and resources to report violence or bullying at your MAIN place of work are:**

Poor Adequate Very Good Don’t Know

**16. How often do other members of the staff at your MAIN place of work (including supervisors and managers) ever behave towards you in a disrespectful, aggressive, or hostile way?**

Daily Weekly Monthly Less Often Never Not Applicable

**17. How often do customers/clients ever behave towards you in a disrespectful, aggressive, or hostile way?**

Daily Weekly Monthly Less Often Never Not Applicable

**PART C: Preferences for different types of jobs**

*Please read the following:*

– You are asked to state which of the two jobs (A or B) is better.

– Everything about the jobs you are comparing is the same, except for the characteristics shown below.

**Please use the following table to answer question 18:**

|  | **Job A** | **Job B** |
| --- | --- | --- |
| **Hourly wage** | 30% higher than provincial minimum wage | 30% higher than provincial minimum wage |
| **Wage Stability** | Fixed for a given period of time | Hourly wage plus bonus based on profits and/or performance |
| **Flexibility** | Weekdays day and evening | Evenings and weekends |
| **Vacation** | No paid vacation time | 21 days per year |
| **Sick Time** | 1.5 days per month | 1.5 days per month |
| **Health Insurance** | Extended (80% of expenses including drugs, vision, dental) | Basic (50% of expenses including drugs) |
| **Workplace Policies** | Respectful workplace policy (non-discriminatory, non-racist, etc.) | Respectful workplace policy (non-discriminatory, non-racist, etc.) |

**18. Which job do you think is better?** Job A Job B

**Please use the following table to answer question 19:**

|  | **Job A** | **Job B** |
| --- | --- | --- |
| **Hourly wage** | 30% higher than provincial minimum wage | Provincial minimum wage |
| **Wage Stability** | Fixed, but contract can end any time | Hourly wage plus bonus based on profits and/or performance |
| **Flexibility** | Rotating shifts | Rotating shifts |
| **Vacation** | 14 days per year | No paid vacation time |
| **Sick Time** | No paid sick leave | 1.0 day per month |
| **Health Insurance** | Extended (80% of expenses including drugs, vision, dental) | Extended (80% of expenses including drugs, vision, dental) |
| **Workplace Policies** | None | Being valued and understood as an employee (ability to provide input, concerns are acknowledged and acted upon by supervisors) |

**19. Which job do you think is better?** Job A Job B

**Please use the following table to answer question 20:**

|  | **Job A** | **Job B** |
| --- | --- | --- |
| **Hourly wage** | 30% higher than provincial minimum wage | Provincial minimum wage |
| **Wage Stability** | Fixed for a given period of time | Fixed for a given period of time |
| **Flexibility** | Evenings and weekends | Weekdays daytime only |
| **Vacation** | No paid vacation time | 21 days per year |
| **Sick Time** | 1.5 days per month | 1.0 day per month |
| **Health Insurance** | None | None |
| **Workplace Policies** | None | Respectful workplace policy (non-discriminatory, non-racist, etc.) |

**20. Which job do you think is better?** Job A Job B

**Please use the following table to answer question 21:**

|  | **Job A** | **Job B** |
| --- | --- | --- |
| **Hourly wage** | Provincial minimum wage | 15% higher than provincial minimum wage |
| **Wage Stability** | Hourly wage plus bonus based on profits and/or performance | Hourly wage plus bonus based on profits and/or performance |
| **Flexibility** | Weekdays day and evening | Evenings and weekends |
| **Vacation** | No paid vacation time | 21 days per year |
| **Sick Time** | 1.0 day per month | No paid sick leave |
| **Health Insurance** | Extended (80% of expenses including drugs, vision, dental) | None |
| **Workplace Policies** | Being valued and understood as an employee (ability to provide input, concerns are acknowledged and acted upon by supervisors) | None |

**21. Which job do you think is better?** Job A Job B

**Please use the following table to answer question 22:**

|  | **Job A** | **Job B** |
| --- | --- | --- |
| **Hourly wage** | 15% higher than provincial minimum wage | Provincial minimum wage |
| **Wage Stability** | Fixed, but contract can end any time | Fixed, but contract can end any time |
| **Flexibility** | Rotating shifts | Weekdays day and evening |
| **Vacation** | No paid vacation time | No paid vacation time |
| **Sick Time** | 1.0 day per month | 1.5 days per month |
| **Health Insurance** | None | None |
| **Workplace Policies** | Respectful workplace policy (non-discriminatory, non-racist, etc.) | None |

**22. Which job do you think is better?** Job A Job B

**Please use the following table to answer question 23:**

|  | **Job A** | **Job B** |
| --- | --- | --- |
| **Hourly wage** | 30% higher than provincial minimum wage | Provincial minimum wage |
| **Wage Stability** | Fixed, but contract can end any time | Fixed, but contract can end any time |
| **Flexibility** | Weekdays daytime only | Weekdays daytime only |
| **Vacation** | 21 days per year | 21 days per year |
| **Sick Time** | 1.0 day per month | No paid sick leave |
| **Health Insurance** | None | Extended (80% of expenses including drugs, vision, dental) |
| **Workplace Policies** | Being valued and understood as an employee (ability to provide input, concerns are acknowledged and acted upon by supervisors) | Respectful workplace policy (non-discriminatory, non-racist, etc.) |

**23. Which job do you think is better?** Job A Job B

**Please use the following table to answer question 24:**

|  | **Job A** | **Job B** |
| --- | --- | --- |
| **Hourly wage** | 15% higher than provincial minimum wage | 30% higher than provincial minimum wage |
| **Wage Stability** | Fixed for a given period of time | Fixed for a given period of time |
| **Flexibility** | Weekdays day and evening | Rotating shifts |
| **Vacation** | 21 days per year | 14 days per year |
| **Sick Time** | 1.5 days per month | 1.0 day per month |
| **Health Insurance** | None | Basic (50% of expenses including drugs) |
| **Workplace Policies** | Being valued and understood as an employee (ability to provide input, concerns are acknowledged and acted upon by supervisors) | Being valued and understood as an employee (ability to provide input, concerns are acknowledged and acted upon by supervisors) |

**24. Which job do you think is better?** Job A Job B

**Please use the following table to answer question 25:**

|  | **Job A** | **Job B** |
| --- | --- | --- |
| **Hourly wage** | Provincial minimum wage | 30% higher than provincial minimum wage |
| **Wage Stability** | Hourly wage plus bonus based on profits and/or performance | Hourly wage plus bonus based on profits and/or performance |
| **Flexibility** | Weekdays day and evening | Weekdays daytime only |
| **Vacation** | 14 days per year | No paid vacation time |
| **Sick Time** | 1.5 days per month | No paid sick leave |
| **Health Insurance** | None | Basic (50% of expenses including drugs) |
| **Workplace Policies** | Respectful workplace policy (non-discriminatory, non-racist, etc.) | None |

**25. Which job do you think is better?** Job A Job B

**Please use the following table to answer question 26:**

|  | **Job A** | **Job B** |
| --- | --- | --- |
| **Hourly wage** | Provincial minimum wage | Provincial minimum wage |
| **Wage Stability** | Hourly wage plus bonus based on profits and/or performance | Fixed, but contract can end any time |
| **Flexibility** | Evenings and weekends | Weekdays day and evening |
| **Vacation** | 14 days per year | 21 days per year |
| **Sick Time** | 1.5 days per month | No paid sick leave |
| **Health Insurance** | Extended (80% of expenses including drugs, vision, dental) | Basic (50% of expenses including drugs) |
| **Workplace Policies** | None | Being valued and understood as an employee (ability to provide input, concerns are acknowledged and acted upon by supervisors) |

**26. Which job do you think is better?** Job A Job B

**PART D: Earnings and Benefits**

The following information will be used to examine the effect of financial issues on your work–life balance.

The information you provide will remain strictly confidential.

**27. What is your current HOURLY wage at your MAIN job?** $CAD____________/Hour
 Don’t Know  Prefer Not to Answer

**28. What are your (approximate) TOTAL earnings from ALL the work you do? (If possible, base this on your last payslip(s) or personal income tax return.)**

|  | **Total from ALL jobs** (Enter annual **or** two-week pay) | | **MAIN job only** (Enter annual **or** two-week pay) | |
| --- | --- | --- | --- | --- |
|  | Annual earnings | Two-week pay period earnings | Annual earnings | Two-week pay period earnings |
| Gross earnings (before tax) |  |  |  |  |
| Net earnings (after tax) |  |  |  |  |

Don’t Know  Prefer Not to Answer

**29. In addition to your TOTAL earnings, did you receive any benefits or salary bonus?** (e.g., health insurance, dental, vision care, pension plan, sales commission)

Yes [if ‘yes’ SKIP Logic to 29a]
No
 Don’t Know  Prefer Not to Answer

**29a. If yes, what type of benefits OR approximate total value of salary bonus?**

|  |  | If you don’t know the answers to these questions or prefer not to answer, select that option from the drop-down menus below |
| --- | --- | --- |
| **Type of benefits:** | [open text field] | Don’t Know Prefer Not to Answer |
| **Approximate total value of salary bonus?** $CAD | [open text field] | Don’t Know Prefer Not to Answer |

**30. What is the total level of financial debt that you currently have because of your education and training? (Include student loan debt, other debt associated with training and living expenses, ongoing course fees) (If zero, write $‘0’)**Total debt . . . . . . . . . . . . . . . . . . . . . . . . . . . . . . . . . . . . . . . . . . . . . . . . . . . . . . . . . . . . . . . . . .. $CAD __________________
 Don’t Know  Prefer Not to Answer

**31. Please indicate the degree to which you agree with the following statement: “Given my current financial situation and prospects, I believe I will have enough to live on when I retire”.**

Strongly Agree Agree Neutral Disagree Strongly Disagree

**32. Please provide any additional comments about your work (optional).**

**We thank you for your time spent taking this survey.
Your response is appreciated.**

You can choose to enter a draw for 1 of 3 gift cards (your choice from e.g., Walmart, Cineplex, iTunes, Tim Hortons) valued at $100 each (see link below). You must provide your email address to enter the prize draw. Your email address will not be linked to your survey responses. Once the online survey has closed (approximately March 1, 2023), a random draw will take place choosing the 3 winners. Gift card(s) will be sent to winners electronically. Your email address will be deleted after the winners are selected for the prize draw.

Draw link: __________________________________________
