# Supplementary material for: The employment preferences of young people in Canada: a discrete choice experiment
Source: BMC Public Health. 2025 Feb 21;25:715. doi: 10.1186/s12889-025-21515-y (PMC11844131; doi:10.1186/s12889-025-21515-y)
Supplement: Supplementary file 2 — Supplementary Material 2. [file 12889_2025_21515_MOESM2_ESM.pdf]

Appendix Table 1: Main Effects Models for Different Samples

	Full model		Women only		Part-timers		Age 18 – 21		BIPOC		> HS		RFTMS	
	Mean	p-value	Mean	p-value	Mean	p-value	Mean	p-value	Mean	p-value	Mean	p-value	Mean	p-value
Wage	0.4559	0.000	0.4123	0.000	0.5394	0.000	0.4568	0.000	0.4546	0.000	0.5761	0.000	0.5347	0.000
**BASE: Fixed for a given period**														
Hourly wage plus bonus	0.5157	0.000	0.4210	0.001	0.5030	0.001	0.6273	0.000	0.5721	0.000	0.6372	0.000	0.4971	0.002
Fixed but contract can end anytime	-0.6052	0.000	-0.5531	0.000	-0.7354	0.000	-0.7408	0.000	-0.6075	0.000	-0.7076	0.000	-0.5950	0.001
**BASE: Rotating shifts**														
Weekdays daytime only	0.1689	0.151	0.1441	0.303	0.1178	0.456	-0.1427	0.379	0.0384	0.760	0.2233	0.141	0.0608	0.728
Weekdays day & evening	-0.1544	0.181	-0.1306	0.327	-0.2526	0.105	-0.0979	0.523	-0.0857	0.503	-0.1379	0.337	-0.1688	0.291
Evenings and weekends	-0.1452	0.262	-0.1526	0.292	-0.0899	0.586	0.1082	0.562	-0.0575	0.644	-0.2734	0.097	-0.1902	0.291
**BASE: No paid vacation**														
21 days per year	0.8749	0.000	0.8429	0.000	0.9090	0.000	0.7393	0.000	0.7640	0.000	1.0461	0.000	0.9778	0.000
14 days per year	0.2296	0.032	0.2310	0.056	0.2464	0.127	0.2291	0.133	0.1589	0.176	0.2865	0.028	0.1949	0.200
**BASE: No paid sick leave**														
1.5 days per month	0.2714	0.001	0.2413	0.009	0.2395	0.025	0.3446	0.004	0.2265	0.009	0.2352	0.023	0.3978	0.002
1.0 days per month	0.1359	0.066	0.1585	0.061	0.1197	0.232	0.0946	0.350	0.1594	0.039	0.2031	0.024	0.0387	0.714
**BASE: No insurance**														
Extended (80%)	1.2612	0.000	1.2366	0.000	1.3049	0.000	1.1104	0.000	1.1897	0.000	1.5354	0.000	1.3568	0.000
Basic (50%)	0.1386	0.060	0.1322	0.132	0.1135	0.254	0.0609	0.563	0.0936	0.240	0.0247	0.782	0.1376	0.217
**BASE: No policy**														
Respectful workplace policy	0.4395	0.001	0.3425	0.016	0.6762	0.001	0.5851	0.002	0.4924	0.001	0.4927	0.004	0.5133	0.007
Being valued as an employee	0.7317	0.000	0.8299	0.000	0.8673	0.000	0.7335	0.000	0.6705	0.000	0.9015	0.000	1.0142	0.000
Constant (Job A)	0.2794	0.003	0.2747	0.009	0.3460	0.007	0.0793	0.540	0.2683	0.007	0.2455	0.026	0.1030	0.428
n (cases)	2,079		1,611		1,278		1,044		1,494		1,593		1,116	
AIC	2,390.0		1,865.0		1,446.7		1,221.7		1,717.1		1,788.2		1,268.7	
BIC	2,549.5		2,017.8		1,592.9		1,362.2		1,867.7		1,940.7		1,411.2	
Pseudo R^2	0.1884		0.1886		0.2121		0.1909		0.1954		0.2147		0.2149	

Appendix Table 2: Willingness to Pay for Different Samples

	Full model	Women	Part-timers	Age 18 – 21	BIPOC	> HS	RFTMS
**BASE: Fixed for a given period**							
Hourly wage plus bonus	0.9347	0.7005	0.5015	1.1248	1.1805	0.9840	0.7464
Fixed but contract can end anytime	-1.5237	-1.6620	-1.7945	-1.8700	-1.4144	-1.3506	-1.2960
**BASE: Rotating shifts**							
Weekdays daytime only	0.0839	0.0120	-0.1982	-0.6023	-0.1460	0.0614	-0.4441
Weekdays day & evening	-0.6253	-0.6541	-0.8848	-0.5044	-0.4190	-0.5656	-0.8735
Evenings and weekends	-0.6053	-0.7076	-0.5832	-0.0532	-0.3569	-0.8009	-0.9135
**BASE: No paid vacation**							
21 days per year	4.3417	4.6488	3.8276	3.7385	3.7106	4.1293	4.0216
14 days per year	2.9263	3.1647	2.5990	2.6217	2.3795	2.8105	2.5574
**BASE: No paid sick leave**							
1.5 days per month	1.4887	1.5548	1.1098	1.7160	1.3469	1.1690	1.5602
1.0 days per month	1.1915	1.3541	0.8878	1.1687	1.1994	1.1134	0.8887
**BASE: No insurance**							
Extended (80%)	5.8366	6.3192	5.0491	4.9949	5.4403	5.3737	5.3320
Basic (50%)	3.3741	3.6404	2.8401	2.6973	3.0291	2.7512	3.0520
**BASE: No policy**							
Respectful workplace policy	3.5329	3.6741	4.1151	4.1675	3.6412	3.2755	3.8166
Being valued as an employee	4.1737	4.8562	4.4694	4.4925	4.0331	3.9853	4.7532
n (cases)	2,079	1,611	1,278	1,044	1,494	1,593	1,116

Appendix Table 3A: Interaction Effects Models

	Part-Time (*n* = 1,242)		Age 18 – 21 (*n* = 1,044)		Women (*n* = 1,611)		RFTMS (*n* = 927)		BIPOC (*n* = 1,494)		HS or less (*n* = 486)	
	Mean	p-value	Mean	p-value	Mean	p-value	Mean	p-value	Mean	p-value	Mean	p-value
**MAIN EFFECTS**												
Wage	0.4424	0.000	0.4458	0.000	0.4411	0.000	0.4562	0.000	0.4422	0.000	0.4657	0.000
**BASE: Fixed for a given period**												
Hourly wage plus bonus	0.5857	0.000	0.3565	0.012	0.5491	0.005	0.4237	0.002	0.3275	0.066	0.5037	0.000
Fixed but contract can end anytime	-0.5010	0.003	-0.3770	0.014	-0.6554	0.002	-0.5652	0.000	-0.5836	0.005	-0.6791	0.000
**BASE: Rotating shifts**												
Weekdays daytime only	0.2375	0.176	0.4489	0.005	0.2934	0.219	0.0516	0.745	0.4288	0.055	0.2457	0.084
Weekdays day & evening	-0.0320	0.847	-0.2085	0.183	-0.3491	0.140	-0.2379	0.113	-0.3158	0.122	-0.1638	0.231
Evenings and weekends	-0.2808	0.170	-0.3220	0.072	-0.0363	0.891	-0.1218	0.475	-0.4394	0.095	-0.2406	0.119
**BASE: No paid vacation**												
21 days per year	0.9165	0.000	0.9132	0.000	0.6183	0.000	0.8950	0.000	1.0344	0.000	0.9033	0.000
14 days per year	0.1359	0.374	0.2188	0.110	0.4218	0.039	0.2084	0.132	0.2703	0.152	0.3702	0.003
**BASE: No paid sick leave**												
1.5 days per month	0.3825	0.002	0.2710	0.010	0.2204	0.144	0.3199	0.003	0.4237	0.004	0.1752	0.061
1.0 days per month	0.1833	0.109	0.1830	0.073	0.1064	0.451	-0.0046	0.963	0.0287	0.838	0.1537	0.069
**BASE: No insurance**												
Extended (80%)	1.4045	0.000	1.3698	0.000	1.1317	0.000	1.2439	0.000	1.2194	0.000	1.3861	0.000
Basic (50%)	0.1649	0.119	0.1280	0.186	0.1744	0.188	0.1871	0.057	0.2231	0.080	0.0970	0.256
**BASE: No policy**												
Respectful workplace policy	0.3143	0.070	0.2890	0.066	0.7629	0.002	0.4657	0.005	0.4112	0.066	0.3985	0.008
Being valued as an employee	0.7059	0.000	0.8072	0.000	0.4745	0.031	0.9730	0.000	0.8210	0.001	0.9186	0.000
Constant (Job A)	0.2778	0.000	0.2665	0.000	0.2733	0.000	0.2847	0.000	0.2920	0.000	0.3135	0.000

Appendix Table 3A: Interaction Effects Models continued

	INTERACTIONS											
* hourly wage plus bonus	-0.1421	0.418	0.2480	0.145	-0.0814	0.688	0.2416	0.171	0.2594	0.184	0.1317	0.538
* fixed but contract can end anytime	-0.1441	0.481	-0.4038	0.046	0.0955	0.683	-0.1332	0.511	-0.0223	0.924	0.1639	0.493
* weekdays daytime only	-0.1589	0.481	-0.6160	0.006	-0.1561	0.559	0.2136	0.346	-0.3784	0.142	-0.3879	0.175
* weekdays day & evening	-0.2093	0.323	0.0829	0.695	0.2057	0.431	0.1968	0.356	0.2176	0.355	0.0549	0.832
* evenings and weekends	0.2210	0.381	0.4328	0.076	-0.1224	0.680	-0.0582	0.813	0.4045	0.173	0.4282	0.154
* 21 days per year	-0.1257	0.389	-0.1447	0.305	0.2696	0.114	-0.0784	0.584	-0.2664	0.113	0.0011	0.995
* 14 days per year	0.1274	0.497	-0.0299	0.869	-0.2711	0.216	0.0406	0.823	-0.0498	0.810	-0.5733	0.012
* 1.5 days per month	-0.1936	0.178	0.0015	0.991	0.0444	0.788	-0.0773	0.587	-0.2134	0.192	0.4355	0.017
* 1.0 days per month	-0.0981	0.490	-0.1091	0.429	0.0393	0.807	0.2583	0.065	0.1359	0.397	-0.1075	0.528
* extended (80%)	-0.3558	0.098	-0.3185	0.130	0.1134	0.650	0.0346	0.869	0.0289	0.905	-0.3002	0.246
* basic (50%)	-0.0678	0.596	-0.0168	0.894	-0.0604	0.684	-0.0882	0.493	-0.1020	0.473	0.3066	0.055
* respectful workplace policy	0.2467	0.246	0.3739	0.075	-0.3632	0.155	0.0232	0.911	0.0515	0.831	0.2537	0.329
* being valued as an employee	0.0722	0.750	-0.0933	0.675	0.2595	0.295	-0.4579	0.040	-0.0635	0.805	-0.3753	0.156
n (cases)	2,079		2,079		2,079		2,079		2,079		2,079	
AIC	2,366.8		2,391.0		2,408.3		2,402.6		2,404.0		2,389.4	
BIC	2,599.2		2,624.1		2,641.4		2,635.8		2,637.2		2,622.5	
Pseudo R^2	0.1914		0.1970		0.1910		0.1930		0.1925		0.1976	

Appendix Table 3B: Interaction Effects Models

	Has benefits (*n* = 513)		On-site (*n* = 1,485)		Min wage (*n* = 423)		EHNPT (*n* = 927)	
	Mean	p-value	Mean	p-value	Mean	p-value	Mean	p-value
**MAIN EFFECTS**								
Wage	0.4491	0.000	0.4424	0.000	0.4482	0.000	0.4573	0.000
**BASE: Fixed for a given period**								
Hourly wage plus bonus	0.5339	0.000	0.4726	0.007	0.5495	0.000	0.6431	0.000
Fixed but contract can end anytime	-0.6366	0.000	-0.7714	0.000	-0.6636	0.000	-0.6761	0.000
**BASE: Rotating shifts**								
Weekdays daytime only	0.1581	0.253	0.2491	0.249	0.1638	0.289	0.2861	0.046
Weekdays day & evening	-0.2416	0.068	-0.0960	0.629	-0.1923	0.191	0.0249	0.868
Evenings and weekends	-0.0521	0.717	-0.3570	0.138	-0.1974	0.233	-0.1384	0.389
**BASE: No paid vacation**								
21 days per year	0.8659	0.000	0.8150	0.000	0.9412	0.000	0.7496	0.000
14 days per year	0.2643	0.031	0.2770	0.123	0.2677	0.050	0.2551	0.047
**BASE: No paid sick leave**								
1.5 days per month	0.2237	0.014	0.1570	0.266	0.3485	0.001	0.2651	0.008
1.0 days per month	0.1061	0.194	0.1134	0.389	0.0673	0.477	0.2716	0.003
**BASE: No insurance**								
Extended (80%)	1.2488	0.000	1.1805	0.000	1.3311	0.000	1.1392	0.000
Basic (50%)	0.1905	0.021	0.1476	0.230	0.2344	0.017	0.2150	0.015
**BASE: No policy**								
Respectful workplace policy	0.5949	0.000	0.4383	0.041	0.4220	0.007	0.5215	0.001
Being valued as an employee	0.8680	0.000	0.7116	0.001	0.8261	0.000	0.5946	0.000
Constant (Job A)	0.3021	0.000	0.2813	0.000	0.3004	0.000	0.2742	0.000

Appendix Table 3B: Interaction Effects Models continued **INTERACTIONS**

* hourly wage plus bonus	-0.0527	0.799	0.0512	0.791	-0.1945	0.408	-0.2773	0.108
* fixed but contract can end anytime	0.0215	0.930	0.2441	0.281	0.4332	0.127	0.1586	0.431
* weekdays daytime only	0.0695	0.783	-0.1392	0.584	0.0610	0.834	-0.3425	0.128
* weekdays day & evening	0.3788	0.108	-0.0770	0.739	0.1646	0.575	-0.3586	0.091
* evenings and weekends	-0.4754	0.124	0.3047	0.272	0.6341	0.065	0.0291	0.905
* 21 days per year	-0.0046	0.979	0.0298	0.853	-0.2412	0.212	0.2673	0.059
* 14 days per year	-0.1036	0.623	-0.0588	0.771	0.0945	0.706	-0.1180	0.516
* 1.5 days per month	0.1759	0.306	0.1543	0.332	-0.2143	0.242	0.0386	0.782
* 1.0 days per month	0.0858	0.600	0.0243	0.875	0.2309	0.204	-0.3691	0.009
* extended (80%)	-0.0208	0.932	0.0715	0.760	0.0799	0.800	0.2140	0.314
* basic (50%)	-0.1320	0.368	0.0031	0.982	-0.1576	0.341	-0.1678	0.193
* respectful workplace policy	-0.5266	0.026	0.0224	0.924	-0.0028	0.992	-0.0925	0.658
* being valued as an employee	-0.3690	0.143	0.0884	0.720	0.2559	0.435	0.3372	0.133
n (cases)	2,079		2,079		2,079		2,079	
AIC	2,393.9		2,411.6		2,022.0		2,391.8	
BIC	2,627.1		2,644.7		2,247.8		2,624.9	
Pseudo R^2	0.1960		0.1899		0.1926		0.1967	

Appendix Table 4: Willingness to Pay for Interaction Effect Models

	Part-Time	Age 18 – 21	Women	Food & Retail	BIPOC	HS or less	Has benefits	On-site	Min wage	EHNPT
**MAIN EFFECTS**										
**BASE: Fixed for a given period**										
Hourly wage plus bonus	1.5154	0.7538	1.0042	0.6186	0.1615	0.7052	0.9601	0.3929	0.9716	1.3338
Fixed but contract can end anytime	-0.9411	-0.8914	-1.7267	-1.5491	-1.8992	-1.8349	-1.6464	-2.4192	-1.7352	-1.5506
**BASE: Rotating shifts**										
Weekdays daytime only	0.3665	0.8240	0.4568	-0.5623	0.2320	0.1868	0.0501	0.1023	-0.1385	1.0033
Weekdays day & evening	-0.2425	-0.6505	-1.0001	-1.1968	-1.4520	-0.6925	-0.8400	-0.6777	-0.9331	0.4321
Evenings and weekends	-0.8051	-0.9053	-0.2907	-0.9424	-1.7315	-0.8574	-0.4181	-1.2677	-0.9444	0.0750
**BASE: No paid vacation**										
21 days per year	4.4504	4.5875	3.7601	4.3803	5.2903	4.6744	4.4453	4.3106	4.7975	3.8357
14 days per year	2.6858	3.0299	3.3144	2.8754	3.5620	3.5296	3.1055	3.0943	3.2946	2.7546
**BASE: No paid sick leave**										
1.5 days per month	2.1437	1.6260	1.2404	1.3925	1.9816	1.0826	1.2324	0.9658	1.7054	1.7532
1.0 days per month	1.6934	1.4286	0.9819	0.6811	1.0882	1.0364	0.9705	0.8672	1.0781	1.7675
**BASE: No insurance**										
Extended (80%)	6.7225	6.4322	5.5270	5.8632	6.0202	6.1616	5.9861	5.6704	6.4632	5.4519
Basic (50%)	3.9204	3.6469	3.3565	3.5467	3.7670	3.3931	3.6294	3.3357	4.0160	3.4310
**BASE: No policy**										
Respectful workplace policy	3.0167	3.1069	4.5355	4.1746	3.7169	3.6844	4.5825	3.5898	3.7264	3.5807
Being valued as an employee	3.9020	4.2693	3.8816	5.2866	4.6436	4.8013	5.1905	4.2075	4.6282	3.7405

Appendix Table 4: Willingness to Pay for Interaction Effect Models continued   INTERACTIONS

* hourly wage plus bonus	-0.7766	0.1610	-0.3933	0.4569	0.5436	0.5410	-0.4155	0.1078	-0.1558	-0.9381
* fixed but contract can end anytime	-0.7811	-1.3010	0.0076	-0.3647	-0.0934	0.6103	-0.2505	0.5439	1.2448	0.0150
* weekdays daytime only	-0.8623	-1.7898	-0.7277	0.5651	-1.0425	-0.9694	-0.2073	-0.5752	1.5506	-1.8404
* weekdays day & evening	-0.9762	-0.2223	0.0925	0.5283	0.3053	-0.0186	0.4815	-0.4345	1.7818	-1.8758
* evenings and weekends	-0.0034	0.5626	-0.6513	-0.0306	0.7281	0.7832	-1.4207	0.4282	2.8294	-1.0280
* 21 days per year	2.0984	1.8232	2.9658	2.1638	1.6332	1.5080	2.2658	2.4702	1.8320	3.1077
* 14 days per year	2.6704	2.0807	1.7399	2.4247	2.1230	0.2745	2.0454	2.2700	2.5810	2.2652
* 1.5 days per month	0.1821	0.7802	1.0312	0.9187	0.3652	2.3461	1.7087	1.3636	0.4868	0.5351
* 1.0 days per month	0.3980	0.5320	1.0195	1.6541	1.1551	1.1800	1.5081	1.0695	1.4801	-0.3564
* extended (80%)	1.7859	1.8931	3.3385	3.0950	3.1627	2.5541	2.8187	3.3321	3.4978	3.5301
* basic (50%)	2.4368	2.5699	2.9443	2.8258	2.8667	3.8573	2.5712	3.1776	2.9679	2.6953
* respectful workplace policy	3.5845	3.9268	1.7469	2.2514	2.8762	3.1124	0.0905	2.9000	3.3434	2.7732
* being valued as an employee	3.1900	2.8789	3.1588	1.1969	2.6161	1.7616	0.4414	3.0492	3.9207	3.7129
